# Muscle adaptations in acute SCI following overground exoskeleton + FES training: A pilot study

**DOI:** 10.3389/fresc.2022.963771

**Published:** 2022-10-13

**Authors:** Kristen Hohl, Andrew C. Smith, Rebecca Macaluso, Matthew Giffhorn, Sara Prokup, Denise R. O’Dell, Lina Kleinschmidt, Jim M. Elliott, Arun Jayaraman

**Affiliations:** ^1^ Max Näder Lab for Rehabilitation Technologies / Outcomes Lab, Shirley Ryan AbilityLab, Chicago, IL, United States; ^2^ Department of Physical Medicine and Rehabilitation, Physical Therapy Program, School of Medicine, University of Colorado, Aurora, CO, United States; ^3^ Department of Physical Therapy, University of Kentucky College of Health Sciences, Lexington, KY, United States; ^4^ Department of Physical Therapy / Human Movement Sciences, Feinberg School of Medicine, Northwestern University, Chicago, IL, United States; ^5^ Northern Sydney Local Health District, The Kolling Institute and Faculty of Medicine and Health, The University of Sydney, St. Leonards, NSW, Australia; ^6^ Department of Physical Medicine / Rehabilitation, Feinberg School of Medicine, Northwestern University, Chicago, IL, United States

**Keywords:** exoskeleton, acute-SCI, ekso®, MRI, muscle

## Abstract

**Objective:**

To evaluate the combined effects of robotic exoskeleton and functional electrical stimulation (FES) training on muscle composition during over-ground gait training in persons with acute spinal cord injury (SCI).

**Design:**

Randomized crossover pilot study.

**Setting:**

Inpatient-rehabilitation Hospital.

**Participants:**

Six individuals with acute SCI.

**Intervention:**

Participants were randomized to either receive training with the Ekso® Bionics exoskeleton combined with FES in addition to standard-of-care or standard-of-care alone.

**Outcome measures:**

The main outcome measures for the study were quantified using magnetic resonance imaging (MRI), specifically, lower extremity muscle volume and intramuscular adipose tissue (IMAT). Static balance and fall risk were assessed using the Berg Balance Scale.

**Results:**

Significant improvements were observed in muscle volume in the exoskeleton intervention group when compared to only standard-of-care (*p* < 0.001). There was no significant difference between the groups in IMAT even though the intervention group saw a reduction in IMAT that trended towards statistical significance (*p* = 0.07). Static balance improved in both groups, with greater improvements seen in the intervention group.

**Conclusions:**

Early intervention with robotic exoskeleton may contribute to improved muscle function measured using MRI in individuals with acute SCI.

## Introduction

There are nearly 18,000 new cases of spinal cord injury (SCI) per year with a range of ∼250,000–360,000 persons currently living with SCI in the United States (1). Gait or walking function is commonly compromised following SCI (1). Current SCI clinical practice guidelines (CPGs) for gait training interventions include over-ground walking, treadmill training, and functional electrical stimulation (FES) (2, 3). FES is also often used during gait training to address motor activation impairments in this population (4). Although the use of robotic exoskeletons is not discussed in the 2020 CPG (5), recent studies have highlighted feasibility and potential to improve gait and mitigate pathological sequelae (e.g., muscle changes) following SCI (6, 7).

Following acute SCI, muscular atrophy occurs rapidly with an increase in the intramuscular adipose tissue (IMAT), particularly in lower extremity musculature (8–10). Large reductions in muscle cross-sectional areas of lower extremity (LE) muscles occur within the early weeks after injury (10–12). IMAT may be up to three times higher in persons with SCI just six weeks after injury when compared to matched uninjured controls and continues to increase in the chronic phase (9). Additionally, individuals with cervical level SCI (C5-7) tend to have a higher percentage of LE IMAT when compared to those with thoracolumbar injury (T12-L2) due to the differences in spasticity (13). The consequences of these changes are vast, influencing functional ambulation, bone health, and increased risk of metabolic diseases and associated mortality (8, 12, 14). Gait training interventions such as treadmill training, FES, and robotic exoskeletons are known to minimize deleterious muscle changes in persons with chronic (status post >1 year) SCI (6, 10, 15–18). However, research studying the impact of these interventions in acute SCI during inpatient stay is still minimal.

One way to minimize early onset of disuse/reduced use muscle atrophy is to mobilize individuals with acute SCI early thereby activating their musculature. However, this process is hard as individuals with acute SCI in an inpatient setting do not tolerate upright standing or walking due to SCI related hemodynamic issues and reduced volitional control. Clinicians using exoskeletons in the inpatient settings have anecdotally suggested to us that individuals with acute SCI are able to tolerate upright walking with an exoskeleton at an early stage compared to standard of care and thus can be used as an early gait training tool. Furthermore, current exoskeletons allow FES devices to synchronize with them thereby timing and stimulating the lower extremity muscles while gait training occurs. Thus, in this pilot study, we evaluated the effects of a smart assist software driven exoskeleton (19) combined with FES training on muscle changes and static balance during over-ground gait training in persons with acute SCI. Using magnetic resonance imaging (MRI), we measured skeletal muscle adaptations prior to and following the training. We hypothesized that a decrease in muscle atrophy, quantified using muscle volume and IMAT will occur following FES and exoskeleton gait training when compared to stand alone standard-of-care in individuals with acute SCI in an inpatient rehabilitation setting.

## Methods

### Participant and protocol details

Individuals with an acute (≤1 month), traumatic SCI admitted to inpatient rehabilitation were recruited to the study. Inclusion criteria for the study were SCI between C7-T11 with American Spinal Injury Association Impairment Scale (AIS) grade A-D, no lower extremity (LE) and upper extremity weight bearing precautions, no open skin wounds where device contacts them, weight under 220lbs (99 kg), height between 5′0″–6′4″ (152 cm–193 cm), no concomitant brain injury, and medical clearance from inpatient physician.

Participants acted as their own controls and were randomized using a computer-generated 1 : 1 allocation to an early or late start of the intervention. If a participant was early start, they received intervention for three weeks and then crossed over to the control group for another three weeks. If they were randomized to late start, they started acute rehab in the control group and then crossed over to the intervention group. This study duration was picked because the average length of stay for patients in this population is 6–8 weeks. MRI images were collected at three different time points: before beginning any acute rehab (Pre), after three weeks of rehabilitation (Mid), and after six weeks of rehabilitation (Post). Since none of the participants in their acute-injury state were able to complete walking/gait outcomes, the Berg Balance Scale, a measure of static balance and fall risk, was assessed at each time point to track clinical progress (20). Participants in the study where unable to perform and complete other clinical outcome measures due to the complexity and acuity of the injury. The Institutional Review board of Northwestern University (Chicago) approved this study and all participants provided written informed consent prior to enrolling.

### Ekso device

Ekso GT™ with SmartAssist [Ekso Bionics, Richmond, California] is a lower limb exoskeleton, which allows for overground walking training with the guidance of a physical therapist. Powered by two rechargeable lithium-ion batteries, hip and knee motors, and an adjustable ankle spring, the device can be programmed to allow for customized assistance. The Ekso GT is approved by the FDA for use in SCI, stroke, and mTBI populations. Step initiation initially occurred *via* “FirstStep” mode, in which the physical therapist controls the stepping action when a participant achieves a balanced weight shift. This mode is used to help a participant with device familiarity and sense of balance. Once this is achieved, participants can progress to “ProStep” mode, where the participant initiates stepping by performing a forward and lateral weight shift target that is configured for each user when their profile is setup on the device. Once these weight shift targets are achieved, the device triggers a step. Alternatively, the participants with incomplete injuries utilized the “ProStep+” mode, where the participant initiates stepping by achieving a lateral weight shift on the stance leg, and then lifting the trailing leg. This allows for participants to actively contribute to swing initiation, if they have the lower extremity strength to do so.

### Interventions

**Figure 1:**
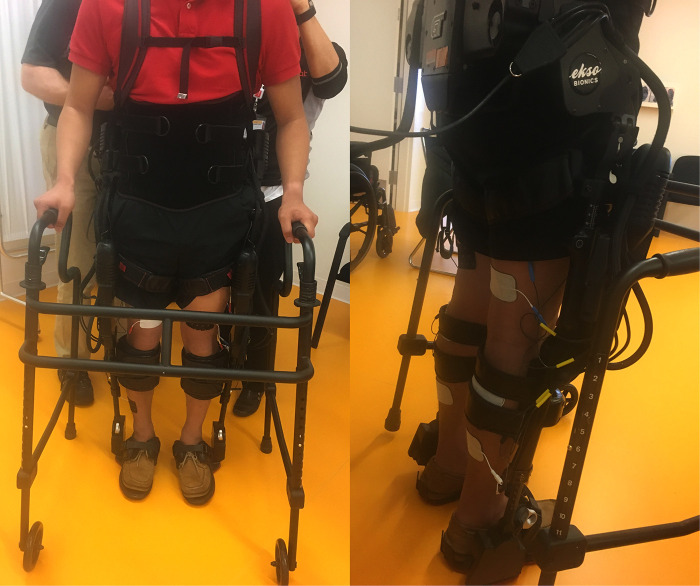
Left: intervention setup with both the exoskeleton and FES from the front. Right: intervention setup with both the exoskeleton and FES from the back.

During the intervention phase/group, training occurred at least 3 days per week as a complement to regular standard-of-care inpatient therapy. Standard of care inpatient therapy occurring during both intervention and control phases included interventions such as transfer training, sitting and standing balance training, mobility training, adaptive equipment education, wheelchair mobility and gait training if appropriate. If scheduling allowed, participants could have up to 5 training sessions per week in addition to their standard-of-care sessions. The first session occurred without additional FES to allow for device fitting and acclimation. All subsequent sessions included FES [Hasomed RehaStim II, Magdeburg, Germany] to bilateral quadriceps, hamstrings, tibialis anterior, and gastrocnemius musculature timed with Ekso's stepping cycle. FES was set at a pulse width 200–300 µs, frequency 35 Hz, and 0-second ramp time. These parameters were chosen to minimize muscular fatigue, maximize training duration and minimize the possibility of autonomic dysreflexia occurring (21, 22). For each muscle, intensity was individually set with the goal to achieve an antigravity muscle contraction, but was occasionally set lower due to patient comfort, cross muscle interference or increased spasticity. Once the pulse parameters were set, stimulation pulses were turned on for 5 min and then off for 2 min for participants without voluntary lower extremity (LE) strength. For those with any LE strength, the training model shifted to 5 min on and 5 min off with decreased robotic assistance to increase volitional effort and provide a greater challenge. During on period, simulation to the muscle groups is timed to the gait cycle. Assessment of LE strength with a manual muscle test was performed weekly during training to ensure all participants received the appropriate FES. All sessions were performed by a licensed physical therapist. Complete setup of intervention training sessions is shown in Figure 1. In the control phase/group of the study, participants received only standard-of-care inpatient therapy.

### Image acquisition

For each time point, using a 3.0 Tesla Siemens Prisma scanner (Siemens, Erlangen, Germany), MRIs were taken of each participants' lower extremity musculature, using a chemical shift based Dixon method to provide fat and water images of the right and left thigh and leg muscles (23–25). A 16-channel body array surface coil was used. Specifics of the MR imaging parameters used are as follows: TR = 7.05 ms, TE1 = 2.46 ms, TE2 = 3.69 ms, flip angle = 12 degrees, bandwidth = 505 Hz/Px, imaging matrix of 448 × 266. A field of view of 237 × 400 mm was used, covering the full thigh or leg anatomy, with 60 slices acquired using a slice thickness of 5 mm.

### Muscle data analyses

Lower extremity muscle data analyses were completed by six trained raters, blinded to clinical presentation and treatment allocation, using medical imaging software OsiriX [Pixmeo SARL, Geneva, Switzerland]. One rater analyzed one participant throughout each time point. Axial images of the lower extremity were used to manually segment the thigh and leg muscles at three time points of data collection. Manual segmentation of lower extremity musculature, for the purpose of muscle quantification, has been shown to have a high level of inter-rater reliability in SCI by the authors and other groups (15, 23).

**Figure 2:**
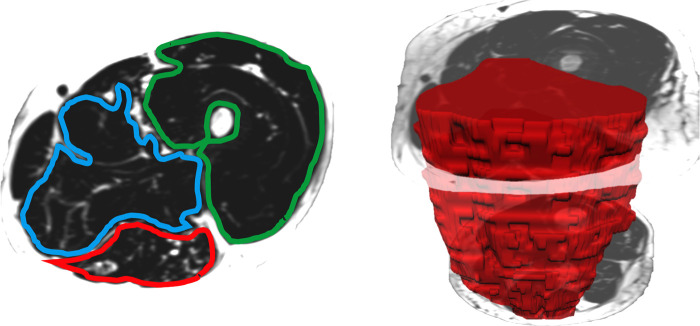
Left: One participants’ fat image of the left thigh, with muscle regions of interest contoured (blue: adductors, green: quadriceps, red: hamstrings). Muscle fat infiltration was observed within each region of interest. Right: Muscle volumes were created by multiplying the cross-sectional area at each slice by the slice thickness (hamstring volume depicted in red).

**Figure 3:**
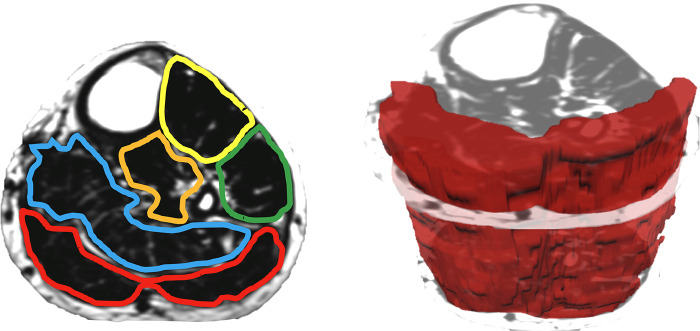
Left: one participants’ fat image of the left leg, with muscle regions of interest contoured (yellow: dorsiflexors, green: peroneus, orange: tibialis posterior, blue: soleus, red: gastrocnemius). Muscle fat infiltration was observed within each region of interest. Right: muscle volumes were created by multiplying the cross-sectional area at each slice by the slice thickness (gastrocnemius volume depicted in red).

When measuring thigh musculature, contouring commenced with the rostral-most slice in which the perineum was not observed and ended when the caudal-most slice was reached. Individual muscle segmentation was completed at each consecutive slice throughout the thigh. For both the right and left thighs, the quadriceps, hamstrings, and adductor muscle groups were segmented (excluding gracilis and sartorius, see Figure 2). The same number of axial slices were used for all three-time points. When measuring leg musculature, each rater began contouring just distal to the knee joint and ended at the last slice where gastrocnemius was still present. For both the right and left legs, the dorsiflexors, gastrocnemius, soleus, posterior tibialis, and peroneus muscle groups were segmented (see Figure 3). The same number of axial slices were used across all three time points.

Regions of interest were created by contouring the muscle group just within the fascial borders, not including the subcutaneous fat or bone, creating a cross-sectional area (CSA) for each muscle at each axial slice (see Figures 2, 3). Individual muscle group volumes were then calculated as the muscle CSA multiplied by the slice thickness (5 mm) using the OsiriX volume calculator tool: Volume_muscle_ = ∑CSA_eachslice_ × 5 mm slice thickness (see Figures 2, 3). IMAT percentages were also calculated using the fat and water images: IMAT% = Fat signal/(Fat signal + Water signal) * 100. All slices were then averaged to produce a single IMAT percentage for that muscle. These calculations were repeated for all muscles at each timepoint.

### Statistical analyses

IBM SPSS Version 28 (Armonk, NY, United States) was used to perform all statistical analyses. A *p*-value of <0.05 was selected to determine statistical significance. A Shapiro-Wilk test was performed to check all data for normality.

For all sixteen muscle groups, percent change scores were calculated for both the control group and for the intervention group (from the time point prior to intervention to the time point following intervention). A non-parametric Wilcoxon Rank Sum test was used to compare the percent change in muscle volume and IMAT% between control and intervention groups as the data was not normally distributed.

## Results

Eight potential participants were recruited for this study. Of the eight, one did not meet the fitting requirements for the exoskeleton and was not enrolled. Of the remaining seven, only six completed the protocol as described above. Demographic information for those who completed the study are presented in Table 1.

**Table 1:** Demographic information for study participants. NIL, neurological injury level; AIS, american spinal injury association impairment scale.

Subject	NIL and AIS grade at baseline	Early or late intervention	Gender	Age (years)	Height (cm)	Weight (kg)	Length of inpatient stay (days)	Time from injury to intervention a start (days)
1	C7 A	Late	M	44	180	88	58	30
2	T9 A	Early	M	21	175	59.4	40	31
3	T10 C	Late	M	28	188	89.8	155	27
4	T11 A	Late	F	36	170	84.4	49	18
5	C8 B	Early	F	19	157	47.6	49	24
6	T5 B	Early	M	50	180	86.2	67	30
**Group mean**				**33**	**175**	**75** **.** **9**	**67**	**27**

### Clinical outcome and training data

**Figure 4:**
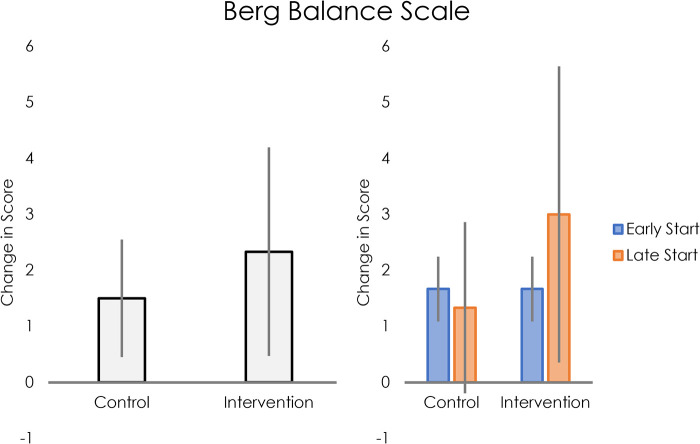
Left: average change in Berg Balance Scale scores for control and intervention groups. Right: Average change in Berg Balance Scale scores for control and intervention groups, separated into early and late intervention start.

Static balance, as measured by the Berg Balance Scale, improved more in the intervention group than the control group (see Figure 4, left panel). For participants who started intervention early, average change in score was the same for the control and intervention phases. For those who received the intervention later, average change in score was greater for the intervention than for control (see Figure 4, right panel).

While all six participants started FES training with 5 min on and 2 min off, participants 5 and 6 gained LE strength and transitioned to 5 min on and 5 min off after 4 and 8 training sessions, respectively. Participants averaged 13 training sessions over the course of the three-week intervention. During these 60–90 min sessions, the average walk time was 28.51 min with an average stimulation time of 20.19 min. Individual breakdown of these and other metrics from training sessions can be found in Table 2.

**Table 2:** Intervention training metrics for study participants.

Participant	Number of training sessions	Mean up time (minutes)	Mean walk time (minutes)	Mean Stim time (minutes)	Mean steps
1	11	40.60	26.80	20.60	726
2	13	37.46	28.85	22.00	919
3	16	45.31	33.13	20.87	1011
4	12	33.92	28.92	21.82	919
5^a^	13	30.77	27.15	17.67	939
6^a^	12	28.67	26.21	18.18	931
**Group mean**	**13**	**36** **.** **12**	**28** **.** **51**	**20** **.** **19**	**907**

^a^ Denotes subject gained LE strength and had a change in FES stimulation parameters.

### Muscle data

**Figure 5:**
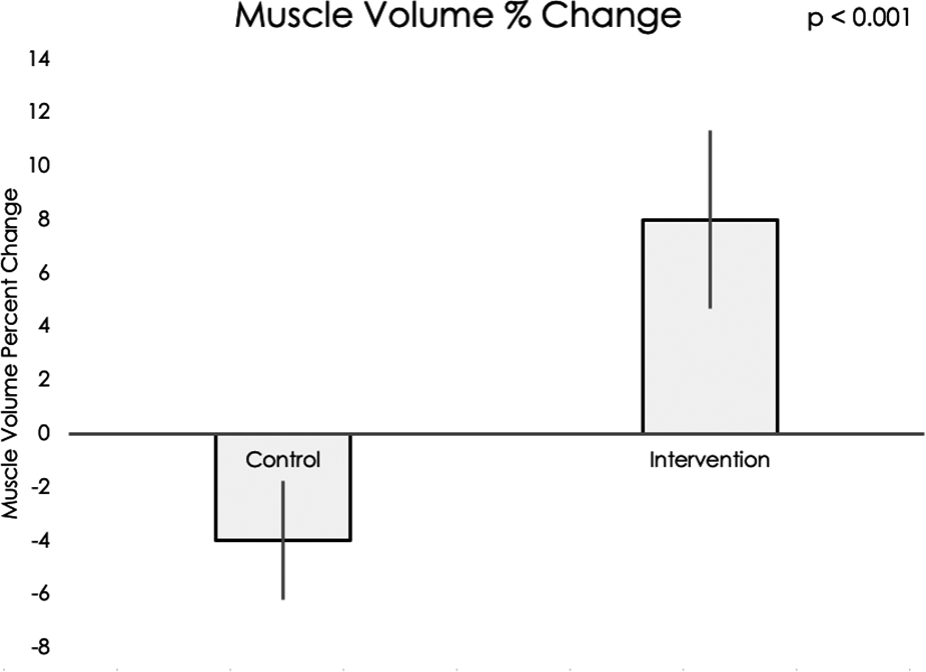
Using a Wilcoxon rank sum test, a significant difference was found in muscle volume percent change in the control group compared to the exoskeleton + FES intervention period (*p* < 0.001).

**Figure 6:**
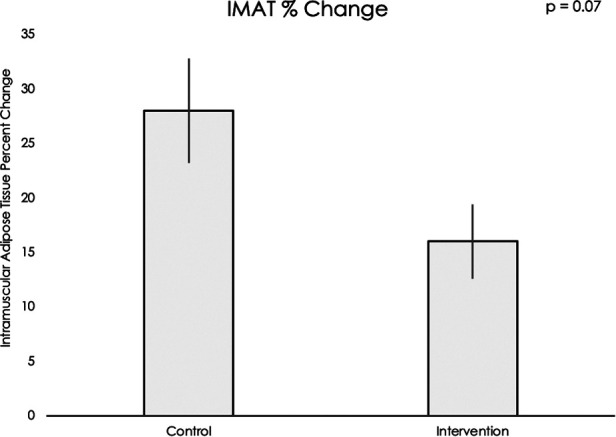
Using a Wilcoxon rank sum test, a trend was found for muscle fat infiltration percent change in the control period compared to the exoskeleton + FES intervention period (*p* = 0.07).

Muscle volume and IMAT percent change data did not meet assumptions for normality, thus nonparametric statistical testing was used. Muscle volumes on an average for all sixteen muscle groups increased significantly during the intervention period compared to the control period (mean difference = 12.20, 95% CI: 4.22, 20.19, *p* < 0.001, see Figure 5). IMAT results demonstrated a trend towards lower percent of fat infiltration during the intervention period compared to the control period (mean difference = 12.30% IMAT, 95% CI: −0.74, 25.35, *p* = 0.07, see Figure 6). Furthermore, no difference was seen in the intervention effect based on time of the intervention (early or late).

## Discussion

Lower extremity muscle groups atrophy immediately and overtime following spinal cord injury (8–10). Interventions such as FES, body-weight supported treadmill training, and overground gait training ameliorate muscle atrophy and improve muscle and motor function following SCI (2–4). In line with these previous studies, our work combined FES and a robotic exoskeleton with overground gait training in individuals with acute SCI (within the first month of injury) in an inpatient rehabilitation setting. Results show that users in the control group experienced muscle atrophy as represented by an average decrease in muscle volume while the intervention group increased muscle volume. Additionally, both groups showed decreased IMAT with the difference between groups trending towards significance. These results indirectly indicate enhanced health and function of lower extremity muscles for better overall functional recovery (14). Though no participant could complete walking outcomes, all showed improvement in static balance over the course of the study. This greater increase in clinical outcome further supports the results found in our image analysis. When differentiating between early and late start groups, the early start group saw the same improvement in both control and intervention phases, while the late start group saw greater improvement during the intervention phase. This suggests that it may be more beneficial for patients to begin with standard-of-care and then transition to exoskeleton and FES training after a certain period of natural recovery.

These results shed light on the impact of robotic exoskeleton and FES training when combined with standard-of-care to help individuals with acute spinal cord injury in the inpatient setting. In the acute phase of recovery, muscle changes occur rapidly through atrophy and fat infiltration (8–10, 12, 14, 26). Early intervention during the acute phase might mitigate the detrimental effects of muscle atrophy and IMAT, thereby increasing muscle metabolic health (16) and providing these patients with potential for better long-term prognosis including initiating early mobility in the right patients. Our study provides a strong base for further investigation of robotic exoskeleton interventions in an inpatient setting and how it impacts patients with acute SCI. Furthermore, many patients after acute-SCI do not tolerate standing or walking due to blood pressure regulation issues. Interestingly, in the current study we identified that all participants were able to immediately tolerate walking with the exoskeleton, thus helping them with early upright mobility. This requires further investigation so we can maximize early mobility in acute SCI.

### Limitations and future work

As a pilot study, this investigation has limitations for clinical application. The sample size was small (*N* = 6) with a heterogeneous representation of patient profiles. These individuals varied in location and severity of injury (according to AIS scale). Subsequent studies should include a larger cohort of participants, as well as homogeneity and organization/grouping according to AIS classification and level. Increasing the numbers of participants may also corroborate previous results of improving muscle volume using FES (27), as our pilot study was likely underpowered to detect these changes. Another limitation of this study is the lack of wash out period. While this choice was made to ensure participants could complete both arms during their inpatient stay, it is possible that carryover effects may have occurred. Future studies may want to assess feasibility of adding a wash out period or having a separate control and intervention group. Further, the use of MRI to assess and monitor changes in muscle may be cost-prohibitive and pose methodological challenges with longitudinal designs. Ultrasound measures of muscle thickness or surface EMG appear promising as a more clinical, cost-friendly approach for lower extremity muscle monitoring following SCI (28).

In addition to addressing limitations of this study, future work could include comparing effects of the combined FES and exoskeleton training with only exoskeleton training, optimizing when a participant should begin receiving combined FES and exoskeleton training, and the addition of other outcomes such as energy expenditure while training, and gait speed.

## Conclusion

Mobility training with robotic exoskeleton and FES as a complement to standard-of-care resulted in reduced muscle atrophy in acute SCI, promoting increases in muscle volume, and maintaining levels of muscle fat infiltration in the lower extremities, when compared to standard-of-care on its own. This is a pilot clinical study and a future larger clinical trial will help generalize the results and standardize the care model.

## Data Availability

The raw data supporting the conclusions of this article will be made available by the authors, without undue reservation.
